# 4*-*Amino-substituted Benzenesulfonamides as Inhibitors of Human Carbonic Anhydrases

**DOI:** 10.3390/molecules191117356

**Published:** 2014-10-28

**Authors:** Kęstutis Rutkauskas, Asta Zubrienė, Ingrida Tumosienė, Kristina Kantminienė, Marytė Kažemėkaitė, Alexey Smirnov, Justina Kazokaitė, Vaida Morkūnaitė, Edita Čapkauskaitė, Elena Manakova, Saulius Gražulis, Zigmuntas J. Beresnevičius, Daumantas Matulis

**Affiliations:** 1 Department of Organic Chemistry, Kaunas University of Technology, Kaunas LT-50254, Lithuania; E-Mails: kestutis.rutkauskas@ktu.lt (K.R.); ingrida.tumosiene@ktu.lt (I.T.); zigmuntas.beresnevicius@ktu.lt (Z.J.B.); 2 Department of Biothermodynamics and Drug Design, Institute of Biotechnology, Vilnius University, Graičiūno 8, Vilnius LT-02241, Lithuania; E-Mails: astzu@ibt.lt (A.Z.); alexeyus1@gmail.com (A.S.); kazokaite@ibt.lt (J.K.); morkunaite@ibt.lt (V.M.); edita.capkauskaite@chf.stud.vu.lt (E.Č.); 3 Department of Physical and Inorganic Chemistry, Kaunas University of Technology, Kaunas LT-50254, Lithuania; E-Mail: kristina.kantminiene@ktu.lt; 4 Institute of Biochemistry, Vilnius University, Mokslininkų 12, Vilnius LT-08862, Lithuania; E-Mail: kazemary@gmail.com; 5 Department of Protein-DNA Interactions, Vilnius University Institute of Biotechnology, Graičiūno 8, Vilnius LT-02241, Lithuania; E-Mails: lena@ibt.lt (E.M.); grazulis@ibt.lt (S.G.)

**Keywords:** benzenesulfonamide, hydrazide, hydrazone, azole, triazene, carbonic anhydrase, fluorescent thermal shift assay, ThermoFluor^®^, isothermal titration calorimetry

## Abstract

A series of *N*-aryl-β-alanine derivatives and diazobenzenesulfonamides containing aliphatic rings were designed, synthesized, and their binding to carbonic anhydrases (CA) I, II, VI, VII, XII, and XIII was studied by the fluorescent thermal shift assay and isothermal titration calorimetry. The results showed that 4-substituted diazobenzenesulfonamides were more potent CA binders than *N*-aryl-β-alanine derivatives. Most of the *N*-aryl-β-alanine derivatives showed better affinity for CA II while diazobenzenesulfonamides possessed nanomolar affinities towards CA I isozyme. X-ray crystallographic structures showed the modes of binding of both compound groups.

## 1. Introduction

Since the discovery of the antibacterial activity of protonsil in 1932 [1], a wide array of aryl- and heteroarylsulfonamides exhibiting a variety of biological activities including antibacterial, antimicrobial, antifungal, anti-HIV, antihelmintic, anti-inflammatory, phytotoxic, cytotoxic, and radiosensitizing properties has been synthesized [2,3,4,5,6,7]. Most of such compounds were secondary sulfonamides. The primary sulfonamides, however, are well known as carbonic anhydrase (CA) inhibitors that are clinically used as antiglaucoma agents, antiephileptic and antiobesity drugs, as well as diuretics [8,9,10,11]. There are 15 CA isoforms, out of which 12 are catalytically active in the human body and several of them are highly over-expressed in various diseases [12,13,14]. Currently used drugs inhibit most of these isoforms with insufficient selectivity, thus causing numerous side effects. The design of isoform-selective CA inhibitors could lead to drugs with fewer side effects compared to the currently used ones [13].

Benzenesulfonamides are the most extensively investigated class of CA inhibitors. Several excellent reviews on CA inhibitors [15,16,17] have discussed the main structural features of such compounds revealing that substituents at the *para*-position of benzenesulfonamides (halogens, acetamido, alkoxycarbonyl moieties, carboxy-, hydrazido-, ureido-, thioureido-, as well as methylamine moieties) are related with good CA-inhibitory properties. To date many CA II-benzenesulfonamide inhibitor crystal structures show similarities in their interaction modes, with the sulfonamide group involved in the coordination of the Zn^2+^ catalytic ion while the phenyl ring establishes several van der Waals interactions with the residues Gln92, Val121, Phe131, Leu198, and Thr200. The substituents on the benzenesulfonamide ring form additional contacts with the hydrophobic and/or the hydrophilic region of the active site, thus contributing favorably to the binding affinity and selectivity.

Novel design of drugs containing benzenesulfonamide scaffolds is often based on the “tail” strategy [17,18], which consists of attaching moieties that provide the desired physico-chemical properties to the sulfonamides possessing free amino groups. The orientation of the tail moiety, the nature of the spacers between the head and the tail moieties, and the presence of polar groups in the tail, strongly influence the potency and selectivity of benzenesulfonamides for the inhibition of CA isoforms [19].

Many benzenesulfonamides were synthesized using sulfanilamide as a starting compound leading to *N*-4-mono-substituted or 4-*N*,*N*-disubstituted derivatives (such compounds were reviewed in [20]). Here we report the synthesis of new *N*-aryl-β-alanine derivatives containing a primary sulfonamide moiety. We investigated these compounds as inhibitors of six carbonic anhydrase isoforms: CA I, II, VI, VII, XII, and XIII.

Triazenes have been extensively studied as agents displaying antitumor activity [21,22]. Several 3,3-dialkyltriazenes bearing sulfonamide groups on the benzene ring were synthesized by Afghani [23] and tested *in vivo* for antitumor activity against mouse lymphoid leukemia, showing no activity. However these compounds were never tested as CAs inhibitors. We synthesized a series of 4-substituted diazobenzenesulfonamides with cyclic amines (pyrrolidine, piperidine, morpholine, and hexamethyleneimine) of various ring sizes as well as with aliphatic amines (dimethylamine and diethylamine) as described by Dabbagh [24] and measured their binding affinity for CA I, II, VI, VII, XII, and XIII, concluding that 4-substituted diazobenzenesulfonamides exhibited significantly higher binding affinity toward tested CAs than *N*-aryl-β-alanine derivatives.

## 2. Results and Discussion

### 2.1. Chemistry

**Scheme 1:**
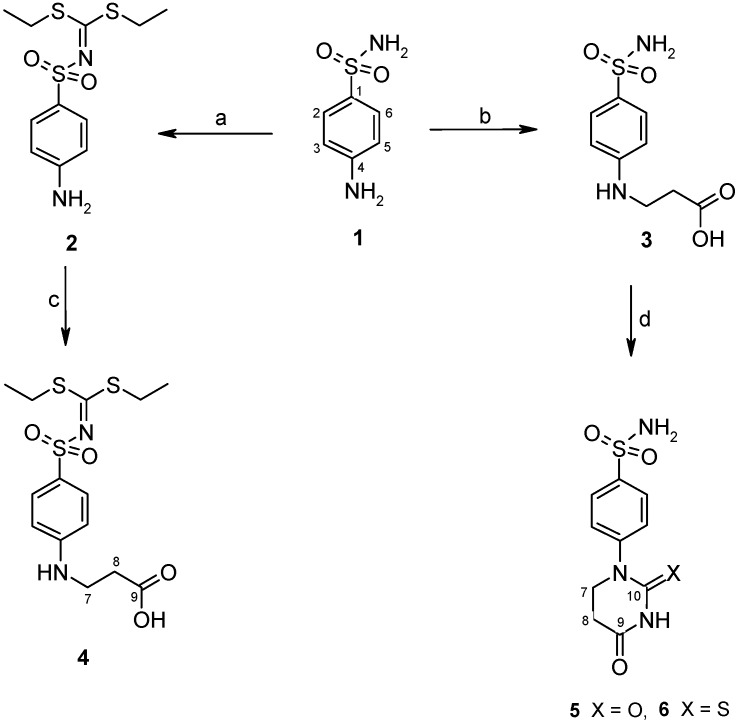
Synthesis route of acid **4** and dihydropyrimidinedione **5** and the thio analogue **6**.

Diethyl 4-aminophenylsulfonylcarbonimidodithioate (**2**) was synthesized from 4-aminobenzene-1-sulfonamide (**1**) by treating it with carbon disulfide and then with ethyl iodide [25] (Scheme 1). *N*-Substituted-β-alanine **3** [26] was obtained in the reaction of **1** with acrylic acid in aqueous solution in the presence of a catalytic amount of hydroquinone, whereas the reaction of **2** with acrylic acid in a mixture of toluene and acetic acid provided 3-[(4-{[bis(ethylthio)-methylene]sulfamoyl}phenyl)amino]propanoic acid (**4**).

*Reagents and conditions*: (**a**) DMSO, CS_2_, C_2_H_5_I, rt, 3 h (90%); (**b**) acrylic acid, hydroquinone, rt, 6 h (69%); (**c**) anhydrous AcOH, acrylic acid, toluene, reflux, 20 h (41%); (**d**) NH_2_CONH_2_ or KCNS, anhydrous AcOH, reflux, 16 h (54%–56%).

**Scheme 2:**
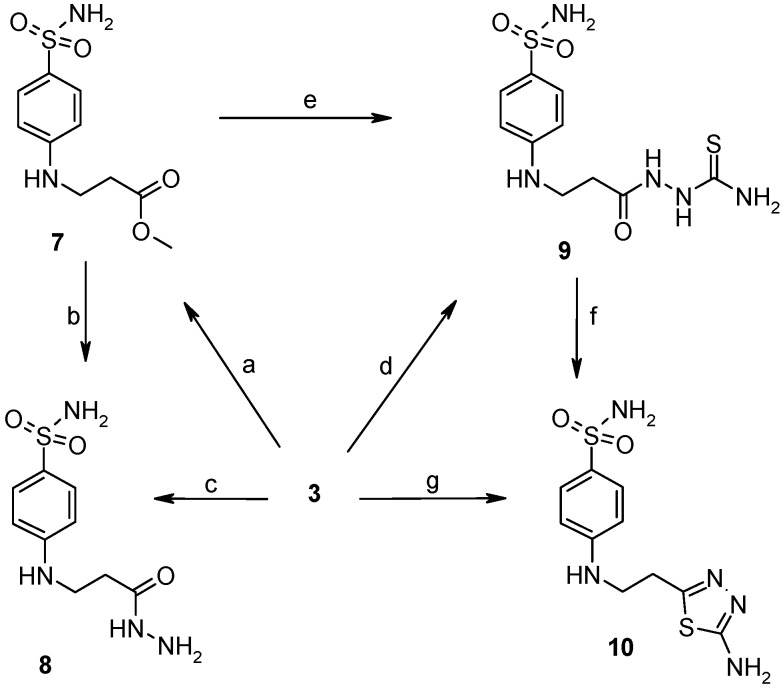
Synthesis route of compounds **7**–**10**.

*N*-Aryl-β-alanines and their esters, upon treatment with carbamide or potassium/sodium thiocyanates in acidic medium, form the N-substituted ureido and thioureido acids which undergo facile cyclization to the corresponding hexahydropyrimidine derivatives [27,28]. Thus, β-alanine **3** was transformed to 4-(2,4-dioxo-1,3-diazinan-1-yl)benzene-1-sulfonamide (**5**) and the corresponding thio analogue **6**. The formation of dihydropyrimidinedione and dihydropyrimidinonethione rings in compounds **5** and **6** has been determined by the presence of the NH group proton singlets at 10.52 ppm for the oxo derivatives and 11.42 ppm for the thio derivatives in the corresponding ^1^H-NMR spectra. Carboxyhydrazides are convenient precursors for the synthesis of heterocyclic compounds [27,28,29,30,31,32]. In the reaction with hydrazine, carboxylic acid esters form hydrazides much easier than acids themselves. Therefore, acid **3** was first converted to ester **7** by heating under reflux in methanol and then the hydrazinolysis reaction of **7** provided 4-{[(2-hydrazinecarbonyl)ethyl]amino}benzene-1-sulfonamide (**8**) (Scheme 2). Heating of acid **3** under reflux with thiosemicarbazide in dioxane in the presence of piperidine or refluxing of ester **7** with thiosemicarbazide gave N-(carbamothioylamino)-3-[(4-sulfamoylphenyl)amino]propanamide (**9**). The latter was cyclized to 4-{[2-(5-amino-1,3,4-thiadiazol-2-yl)ethyl]amino}benzene-1-sulfonamide (**10**) under the action of conc. H_2_SO_4_ at room temperature [30,31]. Compound **10** was also prepared directly from β-alanine **3** by treating it with thiosemicarbazide in sulfuric acid at room temperature followed by the stirring of the reaction mixture at 70 °C. Singlets of the NHNH group protons, characteristic of semicarbazide **9**, observed at 8.67 and 9.10 ppm, are absent in the ^1^H-NMR spectrum of **10** where the resonance of SCNH_2_ group protons is at 7.28 ppm, whereas the protons of CSNH_2_ group gave broad singlet at 4.48 ppm in the ^1^H-NMR spectrum for **9**.

*Reagents and conditions*: (**a**) MeOH, H_2_SO_4_, reflux, 18 h (94%); (**b**) propan-2-ol, NH_2_NH_2_ H_2_O, reflux, 5 h (91%); (**c**) toluene, NH_2_NH_2_ H_2_O, reflux, 5 h (64%); (**d**) dioxane/piperidine, NH_2_CSNHNH_2_, reflux, 16 h (78%); (**e**) MeOH, NH_2_CSNHNH_2_, rt, 3 h (68%); (**f**) H_2_SO_4_, rt, 15 h (34%); (**g**) H_2_SO_4_, NH_2_CSNHNH_2_, 70 °C, 3 h (28%).

**Scheme 3:**
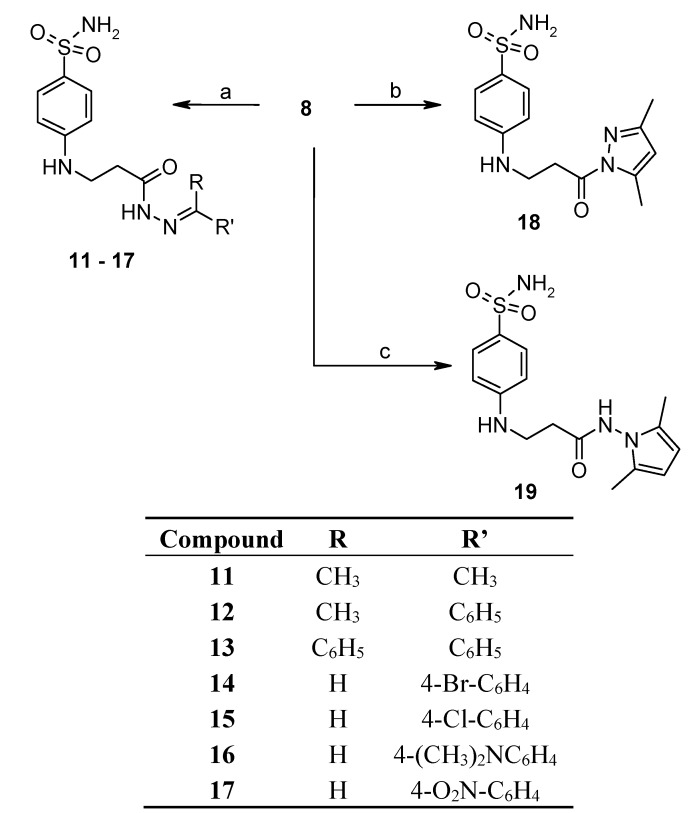
Synthesis route of hydrazones **11**–**17** and compounds **18** and **19**.

The reaction of hydrazide **8** with monocarbonyl compounds provided the corresponding hydrazones (Scheme 3). Thus, the hydrazones **11**−**13** were synthesized in its reactions with acetone, acetophenone and benzophenone, whereas reactions with the substituted benzenecarbaldehydes provided the corresponding benzylidene derivatives **14**−**17**. The presence of the amide group determines the splitting of resonances in ^1^H- and ^13^C-NMR spectra owing to the restricted rotation around the amide bond. The ^1^H- and ^13^C-NMR spectra of **11**−**17** do not reveal the existence of geometrical isomers. Therefore, it has been concluded that in DMSO-*d*_6_ solution these compounds exist as a mixture of *E*/*Z* isomers, where the *Z* isomer predominates because of hindered rotation around the CO-NH bond. Five-membered heterocylic compounds are synthesized from acid hydrazides and aliphatic diketones [29]. Thus, 4-{[3-(3,5-dimethyl-1*H*-pyrazol-1-yl)-3-oxopropyl]amino}benzene-1-sulfonamide (**18**) was obtained from hydrazide **8** by refluxing it with 2,4-pentanedione in propan-2-ol in the presence of conc. HCl. Reaction at reflux temperature of hydrazide **8** with 2,5-hexanedione in the presence of acetic acid provided 4-({2-[*N*-(2,5-dimethyl-1*H*-pyrol-1-yl)-3-[(sulfamoylphenyl)amino] propanamide (**19**) in 10 min. Carbon resonances assigned to the CH_3_ groups in pyrrole moiety are observed at 10.91 ppm in the ^13^C-NMR spectrum of **19**, whereas resonances at 102.84 ppm and 126.69 ppm confirm the existence of a pyrrole ring in this compound.

*Reagents and conditions*: (**a**) MeOH, ketone, reflux, 4 h or propan-2-ol, benzenecarbaldehyde, reflux, 3 h (59%–95%); (**b**) propan-2-ol/HCl, 2,4-pentanedione, reflux, 5 h (49%); (**c**) propan-2-ol/AcOH, 2,5-hexanedione, reflux, 10 min (71%).

**Scheme 4:**
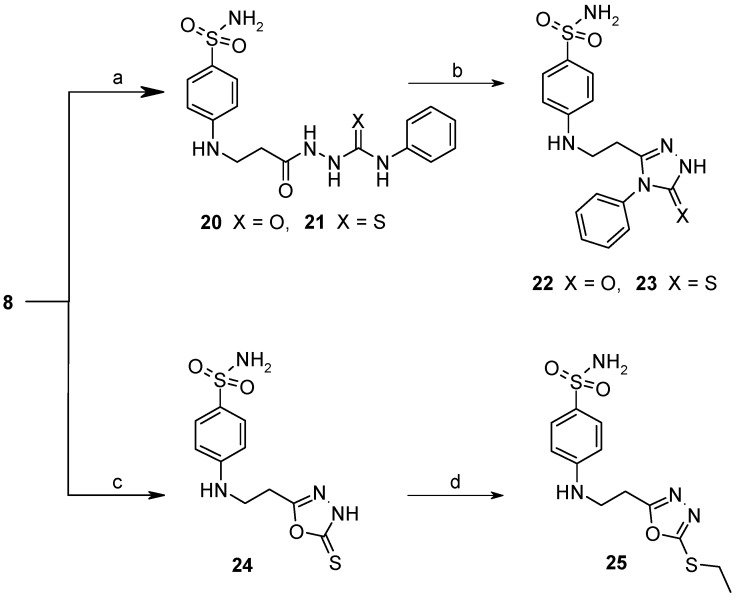
Synthesis route of triazoles **22** and **23** and oxadiazoles **24** and **25**.

The reaction of hydrazide **8** with phenyl isocyanate or phenyl isothiocyanate in propan-2-ol provided *N*-[(phenylcarbamoyl)amino]-3-[(4-sulfamoylphenyl)amino]propanamide (**20**) and its thio analogue **21** (Scheme 4). Depending on the reaction medium, triazoles or oxadiazoles/thiadiazoles can be formed in the cyclization reaction of semicarbazides. Thus, heating under reflux of semicarbazide (**20**) in 20% aqueous NaOH solution with subsequent acidification of the reaction mixture with HCl provided 4-{[2-(5-oxo-4-phenyl-4,5-dihydro-1*H*-1,2,4-triazol-3-yl)ethyl]amino}benzene-1-sulfonamide (**22**) in 68% yield. Likewise, its 5-thio analogue **23** was synthesized from thiosemicarbazide **21**.

When hydrazide **8** was treated with potassium xanthogenate prepared *in situ* from CS_2_ and KOH in ethanol, 4-{[2-(5-sulfanylidene-4,5-dihydro-1,3,4-oxadiazol-2-yl)ethyl]amino}benzene-1-sulfonamide (**24**) was obtained. In the ^1^H-NMR spectrum of **24**, a singlet attributed to a NH proton in oxadiazole-2-thione moiety is observed at 13.76 ppm. 2-Thioxo-2,3-dihydro-3,4-oxadiazoles in reactions with alkyl halides form *S*-alkyl derivatives [32]. Thus, 4-({2-[5-(ethylsulfanyl)-1,3,4-oxadiazol-2-yl]ethyl}-amino)benzene-1-sulfonamide (**25**) was synthesized from oxadiazole-2-thione **24** under treatment with iodoethane under alkaline conditions. In the ^1^H-NMR spectrum of the *S*-substituted derivative **25**, the singlet of the NH group proton in the heterocyclic moiety is absent in comparison with the spectrum of **24** (13.76 ppm), whereas resonances of the CH_3_CH_2_ group protons are observed at 1.38 ppm and 3.22 ppm, respectively.

*Reagents and conditions*: (**a**) MeOH, PhNCO or PhCNS, reflux, 5 min or 4 h (80%–88%); (**b**) 20% NaOH, reflux, 4 h (68%–88%); (**c**) EtOH, CS_2_, KOH, reflux, 20 h (62%); (**d**) MeOH, TEA, C_2_H_5_I, 30 °C, 24 h (72%).

### 2.2. Binding Studies

The synthesized compounds could be arranged into two groups. The first group of compounds is the *N*-aryl-β-alanine derivatives containing the primary sulfonamide group **3**–**25** (except in the compound **4** which is a tertiary sulfonamide) and the second group of compounds—diazobenzenesulfonamides **26**–**33**. Compounds such as benzenesulfonamide (BSA) and acetazolamide (AZM) were used as controls. The binding affinity of these compounds to six CA isoforms I, II, VI, VII, XII, and XIII was determined by the fluorescent thermal shift assay (FTSA) and confirmed for two key compounds **18** and **31** by the isothermal titration calorimetry (ITC). The dissociation constants (*K_d_*) of these compounds for the six CA isoforms are listed in Table 1.

Most *N*-aryl-β-alanine derivatives **3**–**25** were weak inhibitors of all tested CAs exhibiting the *K_d_* higher than 1 µM. Only three compounds **5**, **6**, and **18** exhibited the *K_d_* for CA II of 0.83, 0.67, and 0.67 µM, respectively. In most cases, the *K_d_* for any CA isoform were in the comparable ranges as for BSA and sulfanilamide (SA). For example, only two compounds **6** and **18** bound CA I with more than four times stronger *K_d_* (1.11 and 1.67 µM, respectively) than BSA (7.14 µM). For other *N*-aryl-β-alanine derivatives, the *K_d_* for CA I were in the range of 5.88–170 µM. Only compound **10** bound CA I 1.7 times more weakly than the SA (*K_d_* were 170 and 100 µM, respectively). Our binding data for SA are quite different from the data reported in [33,34]. The previously reported inhibition constant *K_i_* for CA I was reported to be 25 µM, for CA II—0.24 µM, for CA VI—0.9 µM, for CA VII—0.07 µM, for CA XII—0.037 µM, and for CA XIII—0.035 µM. However, Murakami and Sly [35] showed that SA was less effective inhibitor of CA I, II, and VI (*K_i_* was 50, 2.0, and 9.9 µM, respectively). Their inhibition constants were more comparable with our *K_d_* values for CA I, II, and VI and differed 2 times for CA I, 6.5 times for CA II and 5.7 times for CA VI. These discrepancies between inhibition and binding data could be attributed to the different conditions used in assays (*i.e.*, temperature and pH).

**Table 1:** Compound dissociation constants for six human CA isoforms, determined by the fluorescent thermal shift assay and isothermal titration calorimetry (values in the brackets) at pH 7.0, 37 °C.

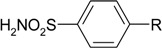							
Compd.	R	Compound Dissociation Constants *K_d_* (µM) for CA Isoforms																
		CA I	CA II	CA VI	CA VII	CA XII	CA XIII
**1**	-NH_2_	100	13.0	56.0	50.0	67.0	100
**3**	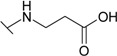	40	12.5	62.5	22.2	9.5	33.3
**4**	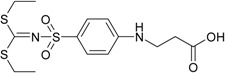 ^a^	>200	40	>200	>200	>200	>200
**5**	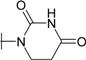	3.85	0.833	12.5	1.54	5.56	8.33
**6**	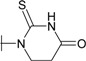	1.11	0.667	8.3	1.61	5.56	2.86
**7**	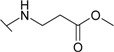	35.7	9.10	71.4	35.7	33.3	58.8
**8**	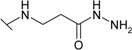	55.6	10.5	62.5	28.6	33.3	76.9
**9**	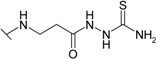	58.8	10.0	50.0	28.6	33.3	76.9
**10**	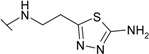	170	20.0	140	100	130	83.0
**11**	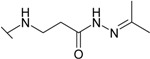	45.5	7.14	71.4	28.6	33.3	50.0
**12**	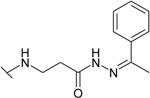	15.4	1.85	25.0	12.5	33.3	5.56
**13**	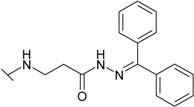	11.5	1.43	25.0	16.7	50.0	4.35
**14**	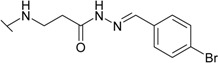	12.5	1.82	7.10	5.88	31.3	7.14
**15**	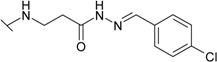	12.5	2.00	6.7	12.5	33.3	8.33
**16**	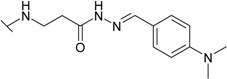	37.0	8.33	50.0	25.0	33.3	55.6
**17**	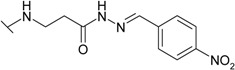	20.0	3.60	25.0	25.0	67.0	50.0
**18**	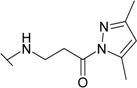	1.67 (0.750)	0.667 (0.454)	27.0 (ND)	3.33 (0.90)	14.3 (ND)	4.00 (1.00)
**19**	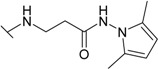	16.7	2.0	25.0	11.1	28.6	6.25
**20**	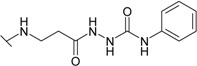	28.6	4.0	7.1	12.5	1.85	18.5
**21**	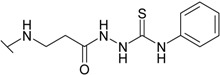	9.09	4.0	8.3	5.56	5.56	9.09
**22**	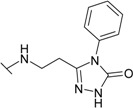	ND	ND	ND	ND	ND	ND
**23**	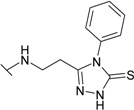	10.0	2.0	11.1	6.67	6.67	10.0
**24**	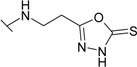	12.5	4.55	33.3	4.55	5.88	26.3
**25**	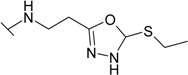	5.88	1.18	28.6	5.60	10.5	3.33
**26**	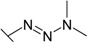	0.100	0.181	13.3	0.769	2.70	0.40
**27**	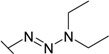	0.0133	0.0714	13.3	0.200	0.588	0.100
**28**	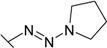	0.0333	0.133	13.3	0.454	1.81	0.167
**29**	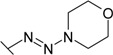	0.0290	0.100	11.8	0.400	3.30	0.222
**30**	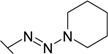	0.00667	0.0625	10.0	0.120	0.770	0.0667
**31**	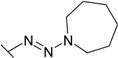	0.0060 (0.022)	0.0435 (0.040)	14.3 (ND)	0.125 (0.192)	0.670 (0.22)	0.0222 (0.078)
**32**	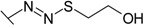	0.0333	0.0667	3.20	0.286	2.00	1.40
**33**	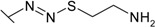	0.500	0.278	8.30	1.00	6.25	2.86
**34**	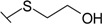 ^b^	0.200 ^b^	0.130 ^b^	4.0	0.170 ^b^	2.50 ^b^	1.40 ^b^
	BSA	7.14	1.79	14.0	6.67	12.50	10.0
	AZM ^c^	1.40	0.017	0.180	0.017	0.133	0.050

ND—not determined due to the limited solubility of the compound; a—entire compound structure, not the R group; b—compound synthesis and binding data described in [36]; c—data taken from [37].

Most *N*-aryl-β-alanine derivatives bound to CA II with higher affinities than to other tested CAs. Interestingly, compound **4**, bearing the tertiary sulfonamide group, bound only to CA II (*K_d_* = 40 µM). Compound **20** showed better binding potency to CA XII (*K_d_* 1.85 µM) than for other isoforms.

**Figure 1:**
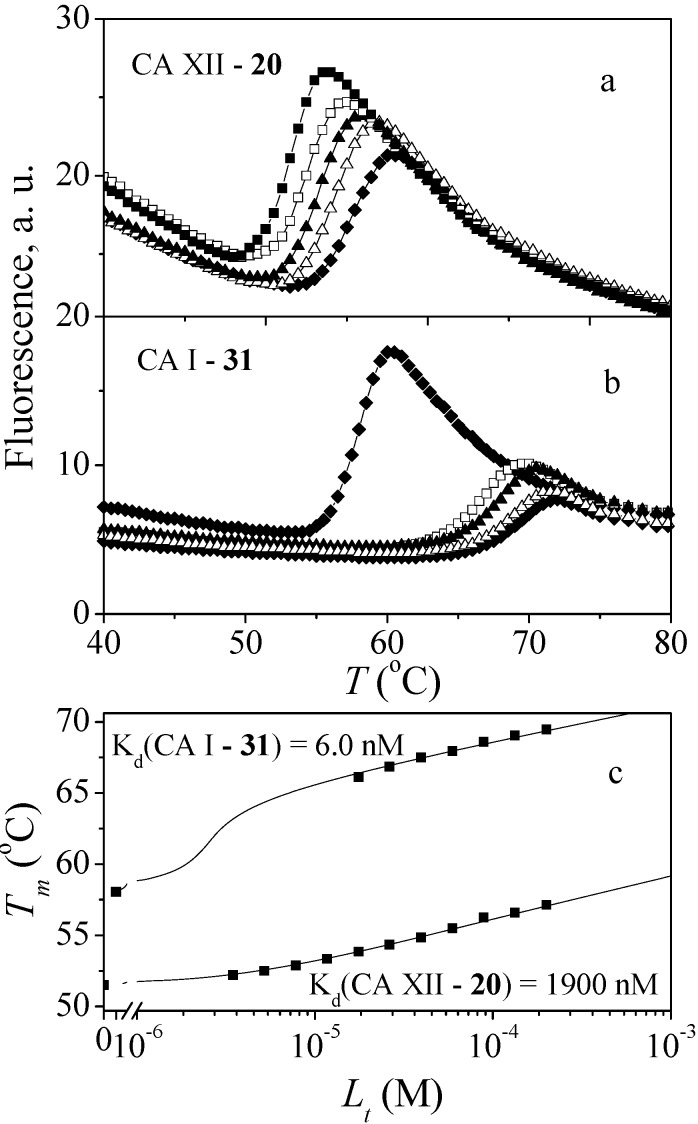
The fluorescent thermal shift assay (FTSA) data for compound **20** binding to CA XII (**a**) and **31** binding to CA I (**b**). Panels on the top (a,b) show the protein melting curves at several added compound concentrations. Panel on the bottom (**c**) shows the dependence of the protein melting temperatures *T_m_* on ligand concentrations. Datapoints are from the experimental values from the upper panels and the curves in panel c are simulated according to the model [38].

**Figure 2:**
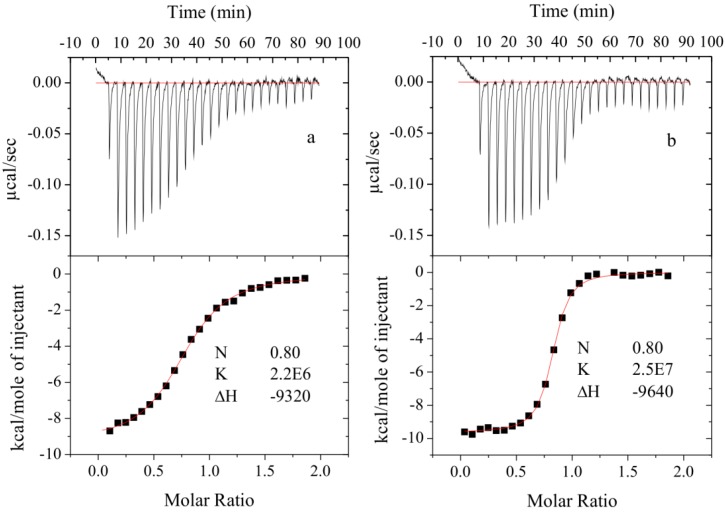
Calorimetric ITC titrations of CA II with **18** (**a**) and **31** (**b**). The experiments were performed at 37 °C in 50 mM phosphate buffer (pH 7.0), 100 mM NaCl and 2% DMSO.

The diazobenzenesulfonamides **26**–**32** were significantly more potent CA inhibitors than *N*-aryl-β-alanine derivatives, especially for CA I and XIII. Figure 1 compares the FTSA data of **31** binding to CA I, as well as **20** binding to CA XII. Compound **31** could be distinguished as the most effective CA I inhibitor in the whole series of tested compounds (*K_d_* = 6 nM), while **20** is selective for CA XII in the group of *N*-aryl-β-alanine derivatives (*K_d_* = 1.85 µM). ITC results were consistent with the FTSA data and confirmed higher binding affinity of diazobenzenesulfonamides rather than the *N*-aryl-β-alanine derivatives (Figure 2).

The increase in binding affinity for CA I and CA XIII was evident with increasing the tail hydrophobicity from dimethylamine group in compound **26** to hexamethyleneimine in **31** (16.7 and 18 times stronger in *K_d_* for CA I and XIII, respectively). The *K_d_* for CA II, VII, and XII did not significantly depend on the hydrophobicity of substituent, whereas such hydrophobic substitutions did not entirely affect the binding affinity to CA VI (*K_d_* were in the range of 10.0–14.3 µM for compounds **26**–**31**). Compounds **27**, **30**, and **31** demonstrated some selectivity for CA I with respect to other isoforms (affinities between at least two isoforms differed at least four-fold). For example, **30** bound to CA I 9.4 times stronger than to CA II, 1500 times stronger than to CA VI, 18 times stronger than to CA VII, 115 times stronger than to CA XII, and 10 times stronger than to CA XIII.

The influence of the diazo group on the binding affinity could be seen comparing the binding data for compounds **32** (bearing the diazo group) and **34** (without the diazo group). As seen in Table 1, the binding affinities are quite similar with the exception of CA I where the binding was about six-fold stronger in the presence than in the absence of the diazo group. However, the influence was negligible for other CAs.

Similar comparison concludes that the presence of amino group diminished the affinity of a compound for CAs. For example, SA bound 5–10 times more weakly than the BSA. Similarly, compound **33** bearing the amino group bound 2–12 times more weakly than compound **32** bearing the hydroxy group.

### 2.3. Crystallography

**Figure 3:**
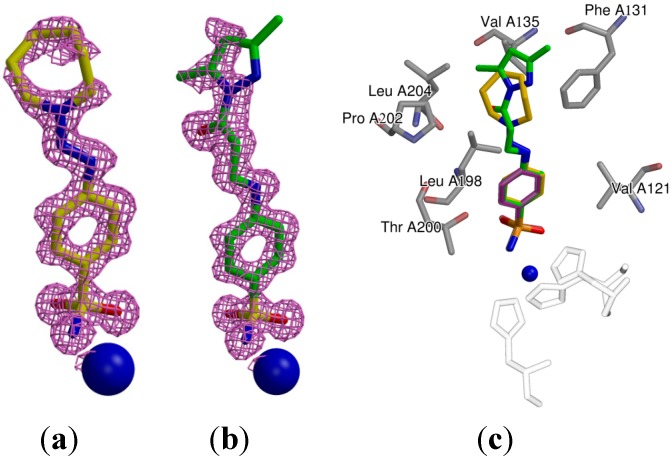
The view of the electron density of the compounds **31** (**a**) and **18** (**b**) located in the active center of CA II as determined by X-ray crystallography. The catalytic Zn(II) atoms are shown as blue spheres. The electron density difference maps are contoured at 2.5σ; (**c**) The superposition of **18** (green), **31** (yellow) and benzenesulfonamide (magenta) from the crystal structure PDB ID 2WEJ [39] bound in the active site of CA II show the identical orientation of the benzene ring. The active site residues of CA II are shown in grey while the histidine residues holding the zinc atom are transparent.

The crystal structures of compounds **31** and **18** bound in the active site of recombinant CA II were solved by X-ray crystallography. Both ligands are well defined in the crystal structures. The difference electron density maps of the compounds calculated from the models omitting the ligand are shown in Figure 3a,b. Benzene rings of **18** and **31** in CA II coincide well with the position of benzenesulfonamide (PDB ID 2WEJ) [39] as shown in Figure 3c. Benzene rings of both ligands make van der Waals contacts with Val121, Leu198, and Thr200. The *para*-substituents of both compounds are fixed between Pro202 and Phe131. Similar orientation was observed for *para*-substituted [(2-pyrimidinylthio)acetyl]-benzenesulfonamides described in [36]. Thus, *para*-groups are located in the CA II active site in mostly hydrophobic environment and contact the residues Phe131, Val135, Leu198, Pro202, and Leu204. The azepane group in *para*-position of **31** is more hydrophobic than of **18** and this probably improves the binding of **31** to CA II as compared to **18** (Table 1). The crystallographic data collection statistics and the PDB IDs are listed in Table 2.

**Table 2:** X-ray crystallographic data collection and refinement statistics. All datasets were collected at 100 K, test set size was 10%.

Protein-Compound	CA II-31	CA II-18
PDB ID	4Q6D	4Q6E
Spacegroup	P2_1_	P2_1_
Unit cell, Å	a = 42.13, b = 41.20, c = 71.81 α = γ = 90°, β = 104.19°	a = 42.24, b = 41.24, c = 72.19 α = γ = 90°, β = 104.30°
Number of chains	1	1
Resolution, Å	39.73–1.12	39.88–1.12
N_ref_ (unique)	87368	85569
R_merge_, (outer shell)	0.076 (0.166)	0.063 (0.193)
I/σ (outer shell)	13.6 (7.0)	14.4 (6.4)
Multiplicity (outer shell)	6.5 (5.1)	6.5 (5.2)
Completeness (%) (outer shell)	95.8 (80.2)	92.8 (66.7)
Number of atoms	2583	2552
Number of solvent molecules	299	321
*R_cryst_* *(R_free_)*	0.128 (0.154)	0.130 (0.158)
RMS bonds/angles	0.025 (2.605)	0.024 (2.362)
Average B-factors (Å^2^)	16.370	14.630
main chain:	13.3	11.7
side chains:	17.4	15.3
solvent:	28.6	25.2
inhibitor:	17.4	19.0

## 3. Experimental Section

### 3.1. General Information

The melting points were determined on a MEL-TEMP (Electrothermal, A Bibby Scientific Company, Burlington, NJ, USA) melting point apparatus and are uncorrected. The ^1^H and ^13^C-NMR spectra were recorded in DMSO-*d*_6_ on a Varian Unity Inova (300 MHz, 75 MHz) and BruckerAvance III (400 MHz, 100 MHz) spectrometers operating in the Fourier transform mode. Chemical shifts (δ) are reported in parts per million (ppm) calibrated from TMS (0 ppm) as an internal standard for ^1^H-NMR, and DMSO-*d*_6_ (39.43 ppm) for ^13^C-NMR. Mass spectra were obtained either on a Waters (Micromass, Milford, MA, USA) ZQ 2000 Spectrometer using ESI (20 eV) ionization or on Bruker maXis UHR-TOF mass spectrometer (Bruker Daltonics, Bremen, Germany) with ESI negative ionization. Samples were introduced using a Waters Acquity UPLC system. Methanol was used as an eluent for sample introduction. The capillary voltage was maintained at +4000 V with the end plate offset at −500 V. Nitrogen was used as the drying and nebulizing gases at a flow rate of 10.0 L/min and a pressure of 2.0 bar, respectively. Elemental analyses (C, H, N) were performed on an Elemental Analyzer CE-440 (Exeter Analytical, Inc., North Chelmsford, MA, USA). The reaction course and purity of the synthesized compounds was monitored by TLC using aluminium plates precoated with silica gel 60 F_254_ (MerckKGaA, Darmstadt, Germany). Synthesis starting reagents were purchased from Sigma-Aldrich (St. Louis, MO, USA) and Fluka (Buchs, Switzerland).

### 3.2. Chemistry

*Diethyl 4-Aminophenylsulfonylcarbonimidodithioate* (**2**): 4-Aminobenzene-1-sulfonamide (**1**, 17.2 g, 0.1 mol) was dissolved in DMSO (50 mL), the solution was cooled down to 0 °C and 30% aqueous NaOH solution (10 mL) was added. The mixture was cooled to 0 °C and stirred at this temperature for 20 min. Afterwards, CS_2_ (6 mL, 0.1 mol) was added dropwise and the stirring was continued for 20 min at 0 °C. 30% aqueous NaOH solution (10 mL) was added again, the mixture was stirred for 20 min and CS_2_ (6 mL, 0.1 mol) was added dropwise again. Temperature of the reaction mixture was raised to room temperature and it was stirred for 1 h. Ethyl iodide (16 mL, 0.2 mol) was added dropwise and the reaction mixture was kept at room temperature for 3 h. Afterwards, it was poured into ice-cold water (200 mL) and conc. HCl was added to pH 2. The precipitate was filtered off and recrystallized from ethanol. Yield 27.4 g (90%); mp 147–148 °C; ^1^H-NMR (300 MHz): δ 1.24 (t, 6H, *J* = 7.2 Hz, 2CH_3_), 3.08 (q, 4H, *J* = 7.2 Hz, *J* = 14.7 Hz, 2CH_2_), 6.10 (s, 2H, NH_2_), 6.63 (d, 2H, *J* = 8.7 Hz, H-3/5), 7.52 (d, 2H, *J* = 8.7 Hz, H-2/6); ^13^C-NMR (75 MHz): δ 13.53 (CH_3_), 27.09 (SCH_2_), 112.46 (C-3/5), 124.87 (C-1), 128.83 (C-2/6), 153.16 (C-4), 180.32 (N=C); ESI–MS: *m*/*z* (%): 305 ([M+H]^+^, 71); Anal. Calcd for C_11_H_16_N_2_O_2_S_3_ (304.452): C, 43.40; H, 5.30; N, 9.20. Found: C, 43.64; H, 5.15; N, 9.33.

*3-[(4-Sulfamoylphenyl)amino]propanoic Acid* (**3**): A mixture of sulfonamide **1** (4.3 g, 25 mmol), water (25 mL), acrylic acid (2 mL, 29 mmol) and catalytic amount of hydroquinone was heated under reflux for 6 h. The reaction mixture was cooled to room temperature, the precipitate formed was filtered off and recrystallized from propan-2-ol. Yield 4.2 g (69%); mp 138–139 °C (138–140 °C [26]); ^1^H-NMR (300 MHz): δ 2.29 (t, 2H, *J* = 6.9 Hz, CH_2_CO), 3.23 (t, 2H, *J* = 6.9 Hz, CH_2_N), 4.21 (br s, 1H, NH), 6.60 (d, 2H, *J* = 9.0 Hz, H-3/5), 6.95 (br s, 2H, NH_2_), 7.49 (d, 2H, *J* = 9.0 Hz, H-2/6); ^13^C-NMR (75 MHz): δ 35.54 (C-8), 39.43 (C-7), 111.59 (C-3/5), 128.25 (C-2/6), 130.74 (C-1), 152.17 (C-4), 174.99 (C-9); ESI–MS: *m*/*z* (%): 245 ([M+H]^+^, 100); Anal. Calcd for C_9_H_12_N_2_O_4_S (244.268): C, 44.25; H, 4.95; N, 11.47. Found: C, 44.65; H, 4.87; N, 11.53.

*3-[(4-{[Bis(ethylthio)methylene]sulfamoyl}phenyl)amino]propanoic Acid* (**4**): A mixture of sulfonamide **2** (3.04 g, 10 mmol), acrylic acid (1.7 mL, 25 mmol), toluene (15 mL) and glacial acetic acid (5 mL) was heated under reflux for 20 h. After solvent evaporation, the residue was dissolved in 10% aqueous NaOH solution (20 mL) and extracted with ethyl ether (2 × 20 mL). The aqueous solution was acidified with acetic acid to pH 5. The precipitate was filtered off and recrystallized from water. Yield 1.54 g (41%); mp 162–163 °C; ^1^H-NMR (300 MHz): δ 1.25 (t, 6H, *J* = 7.2 Hz, 2 CH_3_), 2.54 (t, 2H, *J* = 6.6 Hz, CH_2_CO), 3.09 (q, 4H, *J* = 7.2 Hz, *J* = 14.7 Hz, 2 SCH_2_), 3.33 (q, 2H, *J* = 6.6 Hz, *J* = 12.3 Hz, CH_2_NH), 6.68 (d, 2H, *J* = 9.0 Hz, H-3/5), 6.73 (t, 1H, *J* = 5.4 Hz, NH), 7.59 (d, 2H, *J* = 9.0 Hz, H-2/6), 12.32 (s, 1H, OH); ^13^C-NMR (75 MHz): δ 13.51 (CH_3_), 28.00 (SCH_2_), 33.26 (C-8), 110.75 (C-3/5), 125.01 (C-1), 128.74 (C-2/6), 152.16 (C-4), 172.85 (C-9), 180.68 (N=C); ESI–MS: calculated for C_14_H_20_N_2_O_4_S_3_ 376.5150. Found (positive ions): 378.1345 [M+2H]^+^.

*4-(2,4-Dioxo-1,3-diazinan-1-yl)benzene-1-sulfonamide* (**5**): A mixture of acid **3** (1.69 g, 4.5 mmol), carbamide (2.51 g, 42 mmol) and acetic acid (25 mL) was heated under reflux for 16 h. Afterwards, conc. HCl was added to pH 3 and the mixture was heated under reflux for additional 4 h. The reaction mixture was neutralized with 10% aqueous Na_2_CO_3_ solution, the precipitate was filtered off and recrystallized from methanol. Yield 0.65 g (54%); mp 256–257° C; ^1^H-NMR (400 MHz): δ 2.75 (t, 2H, *J* = 6.6 Hz, CH_2_CO), 3.88 (t, 2H, *J* = 6.6 Hz, CH_2_N), 7.38(s, 2H, NH_2_), 7.54 (d, 2H, *J* = 8.4 Hz, H-3/5), 7.84 (d, 2H, *J* = 8.4 Hz, H-2/6), 10.52 (s, 1H, NH); ^13^C-NMR (75 MHz): δ 30.87 (C-8), 44.06 (C-7), 124.82 (C-3/5), 126.02 (C-2/6); 140.63 (C-1), 144.72 (C-4), 151.95 (C-9), 170.41 (C-10); ESI–MS: *m*/*z* (%): 270 ([M+H]^+^, 85); Anal. Calcd for C_10_H_11_N_3_O_4_S (269.277): C, 44.60; H, 4.12; N, 15.60. Found: C, 45.00; H, 4.38; N, 15.48.

*4-(4-Oxo-2-sulfanylidene-1,3-diazinan-1-yl)benzene-1-sulfonamide* (**6**): A mixture of acid **3** (2.44 g, 10 mmol), potassium thiocyanate (2.42 g, 25 mmol) and acetic acid (15 mL) was heated under reflux for 16 h. Afterwards, conc. HCl was added to pH 2 and the mixture was heated under reflux for additional 4 h. The work-up procedure was analogous to the one for **5**. Yield 1.60 g (56%); mp 217–218 °C; ^1^H-NMR (300 MHz): δ 2.85 (t, 2H, *J* = 6.9 Hz, CH_2_CO), 3.96 (2H, t, *J* = 6.9 Hz, CH_2_N), 7.47 (s, 2H, NH_2_), 7.58 (d, 2H, *J* = 8.7 Hz, H-3/5), 7.89 (d, 2H, *J* = 8.7 Hz, H-2/6), 11.42 (s, 1H, NH); ^13^C-NMR (75 MHz): δ 30.31 (C-8), 48.48 (C-7), 126.56 (C-3/5), 127.83 (C-2/6), 142.74 (C-1), 147.63 (C-4), 166.94 (C-9), 179.52 (C-10); ESI–MS: calculated for C_10_H_11_N_3_O_3_S_2_ 285.3430. Found (positive ions): 286.0431 [M+H]^+^.

*Methyl-3-[(4-sulfamoylphenyl)amino]propanoate* (**7**): A mixture of acid **3** (6.1 g, 25 mmol), methanol (20 mL) and conc. H_2_SO_4_ (0.3 mL) was heated under reflux for 18 h. After solvent evaporation, water (25 mL) was added to the residue and the obtained solution was neutralized with 10% aqueous Na_2_CO_3_ solution to pH 8–9. The precipitate was filtered off and recrystallized from propan-2-ol. Yield 6.1 g (94%); mp 76–77 °C; ^1^H-NMR (300 MHz): δ 2.61 (t, 2H, *J* = 6.9 Hz, CH_2_CO), 3.36 (t, 2H, *J* = 6.9 Hz, CH_2_NH), 3.63 (s, 3H, CH_3_O), 6.38 (br s, 1H, NH), 6.65 (d, 2H, *J* = 8.7 Hz, H-3/5), 6.96 (br s, 2H, NH_2_), 7.53 (d, 2H, *J* = 8.7 Hz, H-2/6); ^13^C-NMR (75 MHz): δ 33.10 (C-8), 38.18 (C-7), 51.38 (CH_3_), 110.77 (C-3/5), 127.35 (C-2/6), 130.25 (C-1), 151.03 (C-4), 171.86 (C-9); ESI–MS: *m*/*z* (%): 259 ([M+H]^+^, 61); Anal. Calcd for C_10_H_14_N_2_O_4_S (258.294): C, 46.50; H, 5.46; N, 10.85. Found: C, 46.62; H, 5.42; N, 10.87.

*4-{[(2-Hydrazinecarbonyl)ethyl]amino}benzene-1-sulfonamide* (**8**): *Method A*. A mixture of ester **7** (2.58 g, 10 mmol), propan-2-ol (15 mL) and hydrazine hydrate (1 mL, 20 mmol) was heated under reflux for 5 h. The precipitate was filtered off and recrystallized from ethanol. Yield 2.34 g (91%). *Method B*. A mixture of acid **3** (2.44 g, 10 mmol), hydrazine hydrate (1.25 mL, 25 mmol) and dry toluene (20 mL) was heated under reflux for 5 h. After solvent evaporation, the residue was recrystallized from ethanol. Yield 1.65 g (64%); mp 145–146 °C; ^1^H-NMR (300 MHz): δ 2.32 (t, 2H, *J* = 6.9 Hz, CH_2_CO), 3.32 (q, 2H, *J* = 6.9 Hz, *J* =12.9 Hz, *CH_2_*NH), 4.27 (br s, 2H, NH*NH_2_*), 6.38 (t, 1H, *J* = 5.7 Hz, NH), 6.63 (d, 2H, *J* = 9.0 Hz, H-3/5), 6.93 (s, 2H, NH_2_), 7.53 (d, 2H, *J* = 9.0 Hz, H-2/6), 9.06 (s, 1H, NHNH_2_); ^13^C-NMR (75 MHz): δ 33.02 (C-8), 38.76 (C-7), 110.76 (C-3/5), 127.29 (C-2/6), 130.12 (C-1), 151.04 (C-4), 169.72 (C-9); ESI–MS: *m*/*z* (%): 259 ([M+H]^+^, 61); Anal. Calcd for C_9_H_14_N_4_O_3_S (258.297): C, 41.85; H, 5.46; N, 21.69. Found: C, 41.40; H, 5.48; N, 21.20.

*N-(Carbamothioylamino)-3-[(4-sulfamoylphenyl)amino]propanamide* (**9**): *Method A*. A mixture of acid **3** (1.22 g, 5 mmol), thiosemicarbazide (0.46 g, 5 mmol), dioxane (8 mL) and piperidine (5 drops) was heated under reflux for 16 h. Precipitate was filtered off and recrystallized from methanol. Yield 1.24 g (78%). *Method B*. A mixture of ester **7** (1.29 g, 5 mmol), thiosemicarbazide (0.46 g, 5 mmol) and methanol (15 mL) was stirred at room temperature for 3 h. After solvent evaporation, ice-cold water (40 mL) was added to the residue. Precipitate was filtered off and recrystallized from methanol. Yield 1.08 g (68%); mp 104–105 °C; ^1^H-NMR (400 MHz): δ 2.32 (t, 2H, *J* = 7.2 Hz, CH_2_CO), 3.31 (q, 2H, *J* = 6.8 Hz, *J* = 12.8 Hz, CH_2_NH), 4.48 (br s, 2H, CSNH_2_), 6.40 (t, 1H, *J* = 5.6 Hz, ArNH), 6.63 (d, 2H, *J* = 8.8 Hz, H-3/5), 6.95 (s, 2H, NH_2_), 7.52 (d, 2H, *J* = 8.8 Hz, H-2/6), 8.67 (s, 1H, NHCO), 9.10 (s, 1H, NHCS); ^13^C-NMR (100 MHz): δ 33.02 (C-8), 38.86 (C-7), 110.72 (C-3/5), 127.33 (C-2/6), 130.05 (C-1), 151.07 (C-4), 169.78 (C-9), 181.08 (C-10); ESI–MS: calculated for C_10_H_15_N_5_O_3_S_2_ 317.3880. Found (positive ions): 340.4524 [M+Na]^+^.

*4-{[2-(5-Amino-1,3,4-thiadiazol-2-yl)ethyl]amino}benzene-1-sulfonamide* (**10**): *Method A*. Semicarbazide **9** (1.59 g, 5 mmol) was dissolved in conc. H_2_SO_4_ (12 mL) at 5 °C and the mixture was stirred at room temperature for 15 h. Afterwards, the reaction mixture was poured into ice-cold water (100 mL) and ammonia was added to pH 8−9. Precipitate was filtered off and recrystallized from propan-2-ol. Yield 0.51 g (34%). *Method B.* Acid **3** (1.22 g, 5 mmol) was dissolved in conc. H_2_SO_4_ (7 mL), the mixture was cooled down to room temperature, thiosemicarbazide (0.46 g, 5 mmol) was added and the reaction mixture was stirred at 70 °C for 3 h. Afterwards, the reaction mixture was poured onto grinded ice (300 mL) and neutralized with ammonia to pH 8−9. Precipitate was filtered off and recrystallized from propan-2-ol. Yield 0.42 g (28%); mp 208–209 °C; ^1^H-NMR (400 MHz): δ 2.66 (t, 2H, *J* = 6.4 Hz, CH_2_C), 3.40 (q, 2H, *J* = 6.4 Hz, *J* = 12.9 Hz, *CH_2_*NH), 6.50 (t, 1H, *J* = 5.6 Hz, NH), 6.67 (d, 2H, *J* = 8.8 Hz, H-3/5), 6.95 (s, 2H, NH_2_), 7.28 (s, 2H, SCNH_2_), 7.54 (d, 2H, *J* = 8.8, H-2/6); ^13^C-NMR (100 MHz): δ 35.88 (C-8), 38.41 (C-7), 110.76, 111.69 (C-3/5), 126.64, 127.36 (C-2/6), 130.04 (C-1), 149.08 (C-4), 160.53 (C-9), 169.87 (C-10); ESI–MS: calculated for C_10_H_13_N_5_O_2_S_2_ 299.3730. Found (positive ions): 300.6866 [M+H]^+^.

#### 3.2.1. General Procedure for Synthesis of Hydrazones **11**–**13**

A mixture of hydrazide **8** (0.62 g, 2.4 mmol) and ketone (3 mmol) in methanol (15 mL) was heated under reflux for 4 h. Precipitate was filtered off and recrystallized from appropriate solvent.

*4-{[2-(N'-Propan-2-ylidenehydrazinecarbonyl)ethyl]amino}benzene-1-sulfonamide* (**11**): Prepared according to the general synthesis procedure from acetone (15 mL) except that no other solvent was used. Yield 0.59 g (82%); mp 216–217 °C (propan-2-ol); ^1^H-NMR (300 MHz): δ 1.86 (s, 3H, =CCH_3_), 1.93 (s, 3H, =CCH_3_), 2.79 (t, 2H, *J* = 7.2 Hz, CH_2_CO), 3.35 (q, 2H, *J* = 7.2 Hz, *J* = 14.3 Hz, CH_2_NH), 6.37–6.51 (m, 1H, ArNH), 6.66 (d, 2H, *J* = 9.0 Hz, H-3/5), 6.94 (s, 2H, NH_2_), 7.53 (d, 2H, *J* = 9.0 Hz, H-2/6), 10.01 (s, 0.4H, NH), 10.11 (s, 0.6H, NH); ^13^C-NMR (75 MHz): δ 16.99, 17.47 (CH_3_), 24.89, 25.09 (CH_3_), 32.17, 33.47 (C-8), 38.05 (C-7), 110.65, 110.72 (C-3/5), 127.30 (C-2/6), 130.03 (C-1), 150.29 (C=N), 151.12 (C-4), 166.78, 172.59 (C-9); ESI–MS: *m*/*z* (%): 299 ([M+H]^+^, 100); Anal. Calcd for C_12_H_18_N_4_O_3_S (298.361): C, 48.31; H, 6.08; N, 18.78. Found: C, 48.87; H, 6.38; N, 19.10.

*4-{(2-[N'-(1-Phenylethylidene)hydrazinecarbonyl]ethyl}amino]benzene-1-sulfonamide* (**12**): Prepared according to the general synthesis procedure from acetophenone (0.35 mL). Yield 0.65 g (75%); mp 185–186 °C (methanol); ^1^H-NMR (300 MHz): δ 2.27 (s, 1H, CH_3_), 2.64 (t, 0.8H, *J* = 6.6 Hz, CH_2_CO), 2.99 (t, 1.2H, *J* = 6.6 Hz, CH_2_CO), 3.43 (q, 2H, *J* = 6.6 Hz, *J* = 12.3 Hz, *CH_2_*NH), 6.50 (t, 1H, *J* = 5.1 Hz, NH), 6.63–6.71 (m, 2H, H-3/5), 6.94 (s, 2H, NH_2_), 7.39–7.45 (m, 3H, H-2'/4'/6'), 7.50–7.58 (m, 2H, H-2/6), 7.76–7.82 (m, 2H, H-3'/5'), 10.41 (s, 0.4H, NH), 10.59 (s, 0.6H, NH); ^13^C-NMR (75 MHz): δ 13.60, 14.05 (=CCH_3_), 32.24, 33.64 (C-8), 38.10, 38.70 (C-7), 110.64, 110.76 (C-3/5), 125.91 (C-2', 6'), 126.19 (C-4'), 127.33 (C-2/6), 128.92, 129.08 (C-3'/5'), 129.97, 130.08 (C-1), 138.18 (C-1'), 151.07 (C-10), 151.14 (C-4), 167.50, 173.51 (C-9); ESI–MS: calculated for C_17_H_20_N_4_O_3_S 360.4310. Found (positive ions): 361.1340 [M+H]^+^.

*4-({2-[N'-(Diphenylmethylidene)hydrazinecarbonyl]ethyl}amino)benzene-1-sulfonamide* (**13**): Prepared according to the general synthesis procedure from benzophenone (0.5 mL) Yield 0.6 g (59%); mp 170–171 °C (methanol); ^1^H-NMR (300 MHz): δ 2.32 (t, 0.8H, *J* = 6.4 Hz, CH_2_CO), 2.49 (t, 1.2H, *J* = 6.4 Hz, CH_2_CO), 3.34 (t, 0.8H, *J* = 6.6 Hz, CH_2_NH), 3.45 (t, 1.2H, *J* = 6.6 Hz, CH_2_NH), 6.39 (s, 0.4H, NH), 6.52 (s, 0.4H, NH), 6.66 (d, 2H, *J* = 6.0 Hz, H-3/5), 6.96 (s, 2H, NH_2_), 7.29 (d, 2H, *J* = 6.0 Hz, H-2/6), 7.35–7.65 (m, 10H, H-Ar'), 9.29 (s, 0.6H, NH), 9.99 (s, 0.4H, NH); ^13^C-NMR (75 MHz): δ 32.14, 33.67 (C-8), 37.98, 38.64 (C-7), 110.70, 110.84 (C-3/5), 126.96 (C-2'/6'), 127.29 (C-4'), 127.37 (C-2/6), 129.47, 129.18 (C-3'/5'), 130.04, 131.72 (C-1), 137.12, 137.72 (C-1'), 151.02 (C-10), 151.12 (C-4), 167.74, 173.53 (C-9); ESI–MS: calculated for C_22_H_22_N_4_O_3_S 422.500. Found (positive ions): 423.5430 [M+H]^+^.

#### 3.2.2. General Procedure for Synthesis of Hydrazones **14**–**17**

A mixture of hydrazide **8** (0.26 g, 1 mmol), and benzenecarbaldehyde (1.2 mmol) in propan-2-ol (15 mL) was heated under reflux for 3 h. Precipitate was filtered off and recrystallized from propan-2-ol.

*4-[(2-{N'-[(4-Bromphenyl)methylidene]hydrazinecarbonyl}ethyl)amino]benzene-1-sulfonamide* (**14**): Prepared according to the general synthesis procedure from 4-bromobenzaldehyde (0.22 g). Yield 0.39 g (91%); mp 218–219 °C; ^1^H-NMR (400 MHz): δ 2.94 (t, 2H, *J* = 6.4 Hz, CH_2_CO), 3.44 (t, 2H, *J* = 6.4 Hz, CH_2_NH), 6.50 (s, 1H, NH), 6.67 (d, 2H, *J* = 8.4 Hz, H-3/5), 6.96 (s, 2H, NH_2_), 7.54 (d, 2H, *J* = 8.4 Hz, H-2/6), 7.58–7.68 (m, 4H, H-Ar'), 7.99 (s, 0.6H, N=CH), 8.14 (s, 0.4H, N=CH), 11.47, (s, 0.6H, NH), 11.53 (s, 0.4H, NH); ^13^C-NMR (100 MHz): δ 31.66, 33.74 (C-8), 38.00, 38.49 (C-7), 110.68, 110.76 (C-3/5), 123.11 (C-4'), 127.37 (C-2/6), 128.81 (C-3'/5'), 130.02, 130.12 (C-1), 131.76 (C-2'), 133.45, 133.55 (C-1'), 141.70, 144.74 (C-10), 151.06, 151.14 (C-4), 167.07, 172.75 (C-9); ESI–MS: calculated for C_16_H_17_BrN_4_O_3_S 425.300. Found (positive ions): 427.0405 [M+2H]^+^.

*4-[(2-{N'-[(4-Chlorophenyl)methylidene]hydrazinecarbonyl}ethyl]amino)benzene-1-sulfonamide* (**15**): Prepared according to the general synthesis procedure from 4-chlorobenzaldehyde (0.17 g). Yield 0.36 g (95%); mp 226–227 °C; ^1^H-NMR (300 MHz): δ 2.94 (t, 2H, *J* = 6.6 Hz, CH_2_CO), 3.42 (t, 2H, *J* = 6.6 Hz, CH_2_NH), 6.50 (s, 1H, NH), 6.68 (d, 2H, *J* = 8.7 Hz, H-3/5), 6.94 (s, 2H, NH_2_), 7.49–7.73 (m, 4H, H-Ar'), 7.92 (d, 2H, *J* = 8.7 Hz, H-2/6), 8.00 (s, 0.6H, N=CH), 8.16 (s, 0.4H, N=CH), 11.47 (s, 0.6H, NH), 11.52 (s, 0.4H, NH); ^13^C-NMR (75 MHz): δ 31.66, 33.73 (C-8), 38.04, 38.52 (C-7), 110.71, 110.77 (C-3/5), 127.34 (C-2/6), 128.82 (C-3'/5'), 129.96 (C-1), 130.05 (C-2'/6'), 134.09 (C-1'), 134.30 (C-4'), 141.58, 144.66 (C-10), 151.01, 151.08 (C-4), 167.03, 172.71 (C-9); ESI–MS: calculated for C_16_H_17_ClN_4_O_3_S 380.8490. Found (positive ions): 383.0898 [M+2H]^+^.

*4-{[2-(N′-{[4-Dimethylamino)phenyl)methylidene}]hydrazinecarbonyl)ethyl]amino}-benzene-1-sulfonamide* (**16**): Prepared according to the general synthesis procedure from 4-(dimethylamine)benzaldehyde (0.18 g). Yield 0.26 g (66%); mp 179–180.5 °C; ^1^H-NMR (400 MHz): δ 2.47 (t, 0.8H, *J* = 6.8 Hz, CH_2_CO), 2.90 (t, 1.2H, *J* = 6.8 Hz, CH_2_CO), 2.97 (s, 6H, 2CH_3_), 3.42 (q, 2H, *J* = 6.9 Hz, *J* = 13.2 Hz, CH_2_NH), 6.47–6.54 (m, 1H, CH_2_*NH*), 6.65–6.81 (m, 4H, Ar-H), 6.96 (s, 2H, NH_2_), 7.41–7.56 (m, 4H, Ar-H), 7.89 (s, 0.6H, N=CH), 8.02 (s, 0.4H, N=CH), 11.12 (s, 0.6H, NH); 11.15 (s, 0.4H, NH); ^13^C-NMR (100 MHz): δ 31.81, 33.74 (C-8), 38.16 (C-7), 39.73 (CH_3_), 110.67, 110.74 (C-3/5), 111.73 (C-3'/5'), 121.50, 121.54 (C-1'), 127.35 (C-2/6), 127.88, 128.25 (C-2'/6'), 129.96, 130.06 (C-1), 143.75, 146.80 (C-10), 151.09, 151.16 (C-4), 151.22, 151.36 (C-4'),166.30, 172.03 C-9); ESI–MS: calculated for C_18_H_23_N_5_O_3_S 389.4720. Found (positive ions): 390.1717 [M+H]^+^.

*4-[(2-{N′-[(4-Nitrophenyl)methylidene]hydrazinecarbonyl]ethyl)amino]benzene-1-sulfonamide* (**17**): Prepared according to the general synthesis procedure from 4-nitrobenzaldehyde (0.18 g). Yield 0.37 g (94%); mp 227–228.5 °C; ^1^H-NMR (400 MHz): δ 2.56 (t, 0.8H, *J* = 6.8 Hz, CH_2_CO), 2.98 (t, 1.2H, *J* = 6.8 Hz, CH_2_CO), 3.44 (q, 2H, *J* = 6.4 Hz, *J* = 12.4 Hz, CH_2_NH), 6.47–6.56 (m, 1H, NH), 6.68 (dd, 2H, *J* = 2.0 Hz, *J* = 8.8 Hz, H-3/5), 6.95 (s, 2H, NH_2_), 7.54 (dd, 2H, *J* = 2.0 Hz, *J* = 8.8 Hz, H-2/6), 7.92 (d, 2H, *J* = 8.8 Hz, H-2'/6'), 8.10 (s, 0.6H, N=CH), 8.28 (d, 2H, *J* = 8.8 Hz, H-3'/5'), 8.31 (s, 0.4H, N=CH), 11.71 (s, 0.6H, NH), 11.76 (s, 0.4H, NH); ^13^C-NMR (100 MHz): δ 31.59, 33.77 (C-8), 37.94, 38.41 (C-7), 110.68, 110.77 (C-3/5), 124.02 (C-3'/5'), 127.37 (C-2/6), 130.14 (C-1), 127.49, 127.85 (C-2'/6'), 140.47, 140.64 (C-1'), 143.51 (C-10), 147.56, 147.70 (C-4'), 151.04, 151.14 (C-4), 167.45, 173.13 (C-9); ESI–MS: calculated for C_16_H_17_N_5_O_5_S 391.4020. Found (positive ions): 392.1143 [M+H]^+^.

*4-{[3-(3,5-Dimethyl-1H-pyrazol-1-yl)-3-oxopropyl]amino}benzene-1-sulfonamide* (**18**): A mixture of hydrazide **8** (0.52 g, 2 mmol), 2,4-pentanedione (0.6 g, 6 mmol), propan-2-ol (8 mL) and conc. HCl (0.1 mL) was heated under reflux for 5 h. Precipitate was filtered off and recrystallized from propan-2-ol. Yield 0.32g (49%); mp 169–170 °C; ^1^H-NMR (300 MHz): δ 2.19 (s, 3H, N=CCH_3_), 2.49 (d, 3H, *J =* 0.9 Hz, N–CCH_3_), 3.34 (t, 2H, *J* = 6.6 Hz, CH_2_CO), 3.46 (t, 2H, *J* = 6.6 Hz, CH_2_NH), 6.49 (br s, 1H, Ar*NH*), 6.20, (q, 1H, *J* = 0.6 Hz, *J* = 20.1 Hz, CH), 6.67 (d, 2H, *J* = 9.0, H-3/5), 6.95 (s, 2H, NH_2_), 7.54 (d, 2H, *J* = 9.0 Hz, H-2/6); ^13^C-NMR (75 MHz): δ 13.44, 14.07 (CH_3_), 34.49 (C-8), 37.74 (C-7), 110.17 (C-3/5), 111.81 (C-11), 127.36 (C-2/6), 130.18 (C-1), 143.22 (C-10), 151.05 (C-12), 151.44 (C-4), 171.70 (C-9); ESI–MS: calculated for C_14_H_18_N_4_O_3_S 322.3830. Found (positive ions): 322.6866 [M]^+^.

*4-({2-[N-(2,5-Dimethyl-1H-pyrol-1-yl)-3-[4-(sulfamoylphenyl)amino]propanamide* (**19**): A mixture of hydrazide **8** (0.65 g, 2.5 mmol), 2,5-hexanedione (0.57 g, 5 mmol), propan-2-ol (10 mL) and acetic acid (0.5 mL) was heated under reflux until precipitate formed (approx. 10 min). Precipitate was filtered off and recrystallized from propan-2-ol. Yield 0.60 g (71%); mp 182–183 °C; ^1^H-NMR (300 MHz): δ 1.98 (s, 6H, 2CH_3_), 2.58 (t, 2H, *J* = 6.6 Hz, CH_2_CO), 3.46 (t, 2H, *J* = 6.6 Hz, CH_2_NH), 5.64 (s, 2H, 2CH), 6.51 (br s, 1H, Ar*NH*), 6.69 (d, 2H, *J* = 8.7 Hz, H-3/5), 6.97 (br s, 2H, NH_2_), 7.55 (d, 2H, *J* = 8.7 Hz, H-2/6), 10.65 (s, 1H, NH); ^13^C-NMR (75 MHz): δ 10.91(CH_3_), 32.77 (C-8), 38.59 (C-7), 102.84 (C-11/12), 110.80 (C-3/5), 126.69 (C-10/13), 127.37 (C-2/6), 130.21 (C-1), 150.96 (C-4), 170.07 (C-10); ESI–MS: calculated for C_15_H_20_N_4_O_3_S 336.4090. Found (positive ions): 359.3265 [M+Na]^+^.

*N-[(Phenylcarbamoyl)amino]-3-[(4-sulfamoylphenyl)amino]propanamide* (**20**): A mixture of hydrazide **8** (0.52 g, 2 mmol), phenyl isocyanate (0.27 mL, 2.5 mmol) and methanol (15 mL) was heated under reflux for 5 min. Precipitate formed in still hot reaction mixture was filtered off and recrystallized from propan-2-ol. Yield 0.61 g (80%); mp 199–200 °C; ^1^H-NMR (300 MHz): δ 2.68 (t, 2H, *J* = 6.9 Hz, CH_2_CO), 3.26 (q, 2H, *J* = 6.9 Hz, *J* = 13.2 Hz, *CH_2_*NH), 6.43 (t, 1H, *J* = 5.4 Hz, NH), 6.67 (d, 2H, *J* = 9.0 Hz, H-3/5), 6.96 (s, 2H, NH_2_), 6.98 (t, 1H, *J* = 7.5 Hz, H-4'), 7.28 (t, 2H, *J* = 7.5 Hz, H-3'/5'), 7.46 (d, 2H, *J* = 7.5 Hz, H-2'/6'), 7.55 (d, 2H, *J* = 9.0 Hz, H-2/6), 8.07, 8.74 (2s, 2H, NHNHCO), 9.78 (s, 1H, NH); ^13^C-NMR (75 MHz): δ 32.83 (C-8), 38.63 (C-8), 110.80 (C-3/5), 118.44 (C-2'/6'), 121.88 (C-4'), 127.35 (C-2/6), 128.60 (C-3'/5'), 130.20 (C-1), 139.49 (C-1'), 151.06 (C-4), 155.30 (C-9), 170.50 (C-10); ESI–MS: calculated for C_16_H_19_N_5_O_4_S 377.4180. Found (positive ions): 378.1345 [M+H]^+^.

*N-[(Phenylcarbamothioyl)amino]-3-[(4-sulfamoylphenil)amino]propanamide* (**21**): A mixture of hydrazide **8** (0.52 g, 2 mmol), phenyl isothiocyanate (0.3 mL, 2.5 mmol) and methanol (15 mL) was heated under reflux for 4 h. The work-up procedure was analoguos to the one for **20**. Yield 0.70 g (88%); mp 206–207 °C; ^1^H-NMR (300 MHz): δ 2.50 (t, 2H, *J* = 6.6 Hz, CH_2_CO), 3.36 (t, 2H, *J* = 6.6 Hz, *CH_2_*NH), 6.41 (t, 1H, *J* = 5.4 Hz, NH), 6.65 (d, 2H, *J* = 8.7 Hz, H-3/5), 6.95 (s, 2H, NH_2_), 7.19 (t, 1H, *J* = 7.5 Hz, H-4'), 7.36 (t, 2H, *J* = 7.5 Hz, H-3'/5'), 7.44 (d, 2H, *J* = 7.5 Hz, H-2'/6'), 7.54 (d, 2H, *J* = 8.7 Hz, H-2/6), 9.59 (s, 2H, NHNHCS), 10.02 (s, 1H, NHAr'); ^13^C-NMR (75 MHz): δ 32.85 (C-8), 39.16 (C-7), 110.78 (C-3/5), 125.91 (C-4'), 126.83 (C-2'/6'), 127.41 (C-2/6), 128.82 (C-3'/5'), 130.20 (C-1), 139.77 (C-1'), 151.83 (C-4), 171.07 (C-9), 180.85 (C-10); ESI–MS: *m*/*z* (%): 394 ([M+H]^+^, 100); Anal. Calcd for C_16_H_19_N_5_O_3_S_2_ (393.484): C, 48.84; H, 4.87; N, 17.80. Found: C, 48.76; H, 5.05; N, 17.99.

*4-{[2-(5-Oxo-4-phenyl-4,5-dihydro-1H-1,2,4-triazol-3-yl)ethyl]amino}benzene-1-sulfonamide* (**22**): A mixture of propanamide **20** (0.38 g, 1 mmol) and 20% aqueous NaOH solution (10 mL) was heated under reflux for 4 h. The reaction mixture was cooled down to room temperature and conc. HCl was added to pH 4. Precipitate formed was filtered off and recrystallized from THF and water mixture. Yield 0.24 g (68%); mp 312–313 °C; ^1^H-NMR (300 MHz): δ 2.66 (t, 2H, *J* = 7.2 Hz, CH_2_CO), 3.26 (q, 2H, *J* = 6.9 Hz, *J* = 13.2 Hz, CH_2_NH), 6.43 (d, 2H, *J* = 9.0 Hz, H-3/5), 6.45 (s, 1H, NH), 6.94 (s, 2H, NH_2_), 7.36–7.61 (m, 7H, H-Ar), 11.77 (s, 1H, NH); ^13^C-NMR (75 MHz): δ 25.47 (C-8), 40.26 (C-7), 110.58 (C-3/5), 127.32 (C-2'/6'), 127.56 (C-2/6), 128.68 (C-4'), 129.45 (C-3'/5'), 130.25 (C-1), 132.82 (C-1'), 144.94 (C-9), 150.67 (C-4), 154.37 (C-10); ESI–MS: calculated for C_16_H_17_N_5_O_3_S 359.4030. Found (positive ions): 382.0942 [M+Na]^+^.

*4-{[2-(4-Phenyl-5-sulfanylidene-4,5-dihydro-1H-1,2,4-triazol-3-yl)ethyl]amino}benzene-1-sulfonamide* (**23**): Prepared from propanamide **21** (0.59 g, 1.5 mmol) by following the same synthesis procedure as for **23** except that 15 mL of 20% aqueous NaOH solution was used. Yield 0.5 g (88%); mp 253–254 °C; ^1^H-NMR (300 MHz): δ 2.68 (t, 2H, *J* = 6.9 Hz, CH_2_CO), 3.29 (q, 2H, *J* = 6.9 Hz, *J* = 13.5 Hz, CH_2_N), 6.42 (d, 2H, *J* = 9.0 Hz, H-3/5), 6.46 (t, 1H, NH), 6.95 (s, 2H, NH_2_), H-2/6), 7.45–7.58 (m, 7H, H-2/6 + Ar'-H), 13.78 (br.s, 1H, NH); ^13^C-NMR (75 MHz): δ 25.01 (C-8), 38.97 (C-7), 110.59 (C-3/5), 127.29 (C-2'/6'), 128.36 (C-4'), 129.42 (C-2/6), 129.46 (C-3'/5'), 130.33 (C-1), 133.59 (C-1'), 150.21 (C-4), 150.53 (C-9), 167.59 (C-10); ESI–MS: calculated for C_16_H_17_N_5_O_2_S_2_ 375.4680. Found (positive ions): 398.0712 [M+Na]^+^.

*4-{[2-(5-Sulfanylidene-4,5-dihydro-1,3,4-oxadiazol-2-yl)ethyl]amino}benzene-1-sulfonamide* (**24**): To a solution of KOH (0.22 g, 4 mmol) in water (2 mL), ethanol (7 mL) was added and CS_2_ (0.36 mL, 6 mmol) was added dropwise. The mixture was stirred at room temperature for 15 min. Next, hydrazide **8** (1.03 g, 4 mmol) dissolved in hot 80% ethanol (30 mL) was added and the mixture was heated under reflux for 20 h. After solvent evaporation, the residue was dissolved in water (20 mL) and conc. HCl was added to pH 3–4. Precipitate was filtered off and recrystallized from propan-2-ol. Yield 0.74 g (62%); mp 132–133 °C; ^1^H-NMR (300 MHz): δ 2.98 (t, 2H, *J* = 6.6 Hz, CH_2_CO), 3.49 (q, 2H, *J* = 6.3 Hz, *J* = 12.3 Hz, CH_2_NH), 6.57 (t, 1H, *J* = 5.7 Hz, NH), 6.67 (d, 2H, *J* = 9.0 Hz, H-3/5), 6.97 (s, 2H, NH_2_), 7.54 (d, 2H, *J* = 9.0 Hz, H-2/6), 13.76 (br s, 1H, NH); ^13^C-NMR (5 MHz): δ 25.71 (C-8), 39.33 (C-7), 111.67 (C-3/5), 128.15 (C-2/6), 131.33 (C-1), 151.40 (C-4), 163.14 (C-9), 178.48 (C-10); ESI–MS: calculated for C_10_H_12_N_4_O_3_S_2_ 300.3570. Found (positive ions): 323.0240 [M+Na]^+^.

*4-({2-[5-(Ethylsulfanyl)-1,3,4-oxadiazol-2-yl]ethyl}amino)benzene-1-sulfonamide* (**25**): A mixture of sulfonamide **24** (0.6 g, 2 mmol), triethylamine (0.42 mL, 3 mmol), ethyl iodide (0.48 mL, 6 mmol) and methanol (20 mL) was stirred at 30 °C for 24 h. Precipitate was filtered off and recrystallized from propan-2-ol. Yield 0.47 g (72%); mp 141–142 °C; ^1^H-NMR (300 MHz): δ 1.38 (t, 3H, *J* = 7.2 Hz, CH_2_*CH_3_*), 3.11 (t, 2H, *J* = 6.6 Hz, CH_2_CO), 3.22 (q, 2H, *J* = 7.2 Hz, *J* = 14.7 Hz, CH_2_CH_3_), 3.53 (q, 2H, *J* = 6.6 Hz, *J* = 12.9 Hz, CH_2_NH), 6.56 (t, 1H, *J* = 6.0 Hz, NH), 6.66 (d, 2H, *J* = 8.7 Hz, H-3/5), 6.96 (s, 2H, NH_2_), 7.53 (d, 2H, *J* = 8.7 Hz, H-2/6); ^13^C-NMR (75 MHz): δ 14.78 (*CH_3_*CH_2_), 24.69 (CH_3_CH_2_), 26.43 (C-8), 39.15 (C-7), 110.82 (C-3/5), 127.34 (C-2/6), 130.48 (C-1), 150.66 (C-4), 163.22 (C-9); 166.04 (C-10); ESI–MS: calculated for C_12_H_16_N_4_O_3_S_2_ 328.4100. Found (positive ions): 351.0556 [M+Na]^+^.

Compounds **26**–**33** were synthesized according to the procedure described previously [23,24] and the compound masses were confirmed by ESI-MS to be as theoretically calculated. The synthesis of compound **34** is described in [39].

### 3.3. Protein Preparation

Expression and purification of CA I, II, VI, VII, XII, and XIII was performed as previously described: CA I and VI in [40], CA II in [36], CA VII and XIII in [41], and CA XII in [42].

### 3.4. Determination of Compound Binding

#### 3.4.1. Fluorescent Thermal Shift Assay

Fluorescent thermal shift assay experiments were performed in a Corbett Rotor-Gene 6000 (QIAGEN Rotor-Gene Q, Sydney, Australia) RT-PCR instrument using the blue channel (excitation 365 ± 20, detection 460 ± 15 nm). Sample volume was 20 µL containing 5–10 µM protein, 0–200 µM ligand, 50 µM solvatochromic dye ANS (8-anilino-1-naphthalene sulfonate), and 50 mM sodium phosphate buffer containing 100 mM NaCl at pH 7.0, with the final DMSO concentration at 2%. The samples were heated at a constant rate of 1 °C/min. Data analysis was performed as previously described [43].

#### 3.4.2. Isothermal Titration Calorimetry

Isothermal titration calorimetry (ITC) experiments were performed using VP-ITC instrument (Microcal, Inc., Northampton, MA, USA) with 5–10 µM protein solution in the cell and 50–100 µM of the ligand solution in the syringe. A typical experiment consisted of 25 injections (10 µL each) within 3 min intervals. Experiments were performed at 37 °C in a 50 mM sodium phosphate buffer containing 100 mM NaCl at pH 7.0, with a final DMSO concentration of 2%, equal in the syringe and the cell.

### 3.5. Crystallography

#### 3.5.1. Crystallization

CA II in 20 mM Hepes buffer pH 7.5 and 50 mM NaCl was concentrated by ultrafiltration to the concentration 50 mg/mL. CA II crystals were obtained by sitting drop vapor diffusion method mixing 4 μL of protein solution with 4 μL of crystallization buffer. Crystallization buffer contained 0.1 M sodium bicine, pH 9.0 and 2.0 M sodium malonate, pH 7.5. Crystals were grown at 20 °C for several weeks. Complexes were prepared by soaking crystals with 0.5 mM solution of ligand in crystallization buffer (prepared by mixing of 50 mM stock solution of ligand in DMSO with crystallization solution).

#### 3.5.2. Data Collection and Structure Determination

Diffraction data were collected at the EMBL beam line P13 at the storage ring PETRAIII (DESY, Hamburg, Germany). Datasets were processed using XDS [44], TRUNCATE [45], and SCALA [46,47]. Molecular replacement was performed using MOLREP [48]. The protein moiety from PDB entry 3HLJ was used as an initial model. Model building and refinement was carried out with COOT [49] and REFMAC [50], respectively. Atomic coordinates of ligands were generated using molecular editor Avogadro [51]. Descriptions of ligand geometry for structure refinement were generated with LIBREFMAC [52]. The data collection and refinement statistics as well as PDB access codes (4Q6D, 4Q6E) are presented in Table 2. Graphic representations of crystal structures are prepared with MOLSCRIPT [53], RASTER3D [54], and BOBSCRIPT [55].

## 4. Conclusions

A series of N-aryl-β-alanine and diazobenzenesulfonamides were synthesized and tested as binders of CA isozymes I, II, VI, VII, the catalytic domain of XII, and XIII. The most potent diazobenzenesulfonamides, possessing hexamethylimine and piperidine groups (compounds **30** and **31**), displayed nanomolar binding affinity toward CA I. The X-ray crystallographic cocrystal structures of compounds **18** and **31** bound to CA II showed that the location of the benzene ring did not depend on the *para*-substituent. The more hydrophobic *para*-substituent of inhibitor **31** improved the binding properties due to additional hydrophobic interactions.
